# A multivariate PCA-based evaluation of betaine, vitamin C, and phytogenic (STRESS-BIOMax) - supplemented drinking water in broilers?

**DOI:** 10.1371/journal.pone.0357659

**Published:** 2026-11-02

**Authors:** Morteza Asghari-Moghadam, Hamid-Reza Behboodi, Mehran Mehri

**Affiliations:** 1 Department of Animal Sciences, Faculty of Agriculture, University of Zabol, Sistan, Iran; 2 Department of Animal and Poultry Physiology, Faculty of Animal Science, Gorgan University of Agricultural Science and Natural Resources and Research and Development Department, Makian Rooyesh Darou Co, Gorgan, Golestan, Iran; University of Life Sciences in Lublin, POLAND

## Abstract

Physiological and metabolic disturbances can impair growth and oxidative balance in broilers. This study evaluated the effects of Stress-BioMax (SBM) supplementation in drinking water (0.00, 0.25, 0.50, and 1.00 mL/L) on growth performance, carcass traits, biochemical indices, oxidative status, and systemic physiological responses. SBM is a drinking-water additive containing betaine, vitamin C, and herbal extracts. Principal component analysis (PCA), quadratic regression, and segmented regression were used to assess dose-dependent responses and identify the optimal supplementation range. SBM significantly improved body weight gain and feed efficiency without affecting feed intake. The 0.50 mL/L dose produced the greatest body weight gain (3082 g) and the lowest feed conversion ratio (1.62; P < 0.001). Carcass and muscle yields increased, while abdominal fat and organ percentages decreased (P < 0.001). Serum protein status and lipid profiles improved, with reduced triglycerides, LDL, VLDL, cholesterol, glucose, bilirubin, and cortisol, alongside increased HDL and triiodothyronine levels (P < 0.001). Antioxidant capacity increased, malondialdehyde concentrations decreased, and hepatic enzyme activities were reduced, indicating improved oxidative balance and liver function. PCA suggested a dose-dependent reduction in the composite physiological burden, with the minimum occurring near 0.62 to 0.66 mL/L. Overall, Stress-BioMax enhanced growth performance, metabolic health, antioxidant status, and physiological resilience in broilers.

## Introduction

Modern broiler chickens have been intensively selected for rapid growth and high feed efficiency, but this has inadvertently increased their susceptibility to metabolic disturbances and oxidative stress [1,2]. Broilers possess exceptionally high metabolic rates, elevated blood glucose, and body temperatures, which predispose them to a state of chronic oxidative challenge even under ostensibly normal rearing conditions [2]. Oxidative stress arises when the production of reactive oxygen species (ROS) outpaces antioxidant defenses, leading to cellular damage, inflammation, and impaired physiological functions. In fast-growing broilers, such an imbalance can manifest as “metabolic syndrome”-like conditions, including dysregulated lipid metabolism, systemic inflammation, and hepatic oxidative injury [1]. These subclinical stressors ultimately compromise growth performance, meat quality, and welfare by impairing nutrient utilization and triggering pathologies (e.g., fatty liver, muscle myopathies) if not properly managed [2]. Therefore, mitigating oxidative stress and supporting metabolic homeostasis have become central goals in improving broiler health and productivity.

Among the diverse array of phytogenics, herbal extracts with antioxidant and anti-inflammatory properties are particularly relevant for stress mitigation. In this context, St. John’s wort (*Hypericum perforatum L.*), lavender (*Lavandula angustifolia*), and lemon balm (*Melissa officinalis*) have attracted attention due to their rich content of biologically active compounds. *Hypericum perforatum* is well known for its principal constituent hypericin, alongside flavonoids and phenolic acids, which confer antioxidant, antimicrobial, and neuromodulatory activities [3].

St. John’s wort (*Hypericum perforatum*), lavender (*Lavandula angustifolia*), and lemon balm (*Melissa officinalis*) have been increasingly explored as phytogenic additives in poultry because of their antioxidant, antimicrobial, and metabolism-modulating properties [4]. In broilers, *Hypericum* extract administered via drinking water (150–250 mg/L) improved growth performance similarly to virginiamycin, while also lowering abdominal fat and improving serum lipid indices (reduced triglycerides, total cholesterol, and LDL, with increased HDL), indicating a beneficial influence on lipid metabolism and energy partitioning [5]. Lavender essential oil, rich in linalool and related terpenes, has likewise been studied as a drinking-water additive, where supplementation (0.4 mL/L) improved body weight and feed conversion and was associated with enhanced antioxidant activity and health-related blood indices in broiler chickens [6]. Lemon balm and its major phenolic compound rosmarinic acid are recognized for antioxidant and anti-inflammatory activities, and experimental evidence in broilers supports its hepatoprotective potential and capacity to reduce oxidative and inflammatory injury, consistent with the biological role of rosmarinic acid in modulating oxidative stress pathways [7]. SBM integrates herbal extracts with betaine and vitamin C, compounds commonly used to support antioxidant defenses and physiological function [3].

Despite the known benefits of individual components like vitamin C, there is limited information on the integrated effects of SBM on the global physiological profile of broilers, particularly through multivariate analysis. Traditional univariate statistics often fail to capture the coordinated response of multiple biological systems to dietary interventions. Principal component analysis (PCA) offers a sophisticated approach to integrate various biomarkers, ranging from thyroid hormones and cortisol to hepatic enzymes and antioxidant capacity, into a single composite index of physiological health [8]. Furthermore, the determination of the “optimal dose” for multi-component supplements remains a subject of debate, as linear responses are rarely observed in complex biological systems. Nutritional studies increasingly use quadratic and segmented regression to find the optimal supplement breakpoint [9].

Given the increasing pressure to optimize bird welfare and productivity simultaneously, it is essential to evaluate whether SBM can modulate the thyroid–adrenal stress axis, enhance antioxidant status, and improve carcass yields in a dose-dependent manner.

SBM has previously been evaluated in broiler chickens exposed to chronic heat stress, where supplementation improved growth performance, antioxidant status, liver enzyme activities, and endocrine responses. However, whether these beneficial effects extend to broilers reared under normal production conditions remains unclear. Moreover, previous studies focused primarily on conventional univariate analyses of individual traits, leaving the integrated physiological response and biologically optimal supplementation level unresolved. Therefore, the present study investigated the effects of SBM supplementation in drinking water on growth performance, carcass characteristics, serum biochemistry, antioxidant status, and endocrine responses of broiler chickens reared under normal conditions. In addition to conventional statistical analyses, principal component analysis (PCA) was used to integrate multiple physiological biomarkers into a composite physiological response index, and quadratic and segmented dose–response models were applied to identify the biologically optimal SBM supplementation level.

## Materials and methods

### Ethics statement

All experimental procedures involving broiler chickens were conducted in accordance with the guidelines of the Iranian Council of Animal Care and were approved by the Research Animal Ethics Committee of the University of Zabol, Iran (Approval No. AEUOZ-2012/2026-BR-23). Birds were observed at least twice daily throughout the 42-day experiment for general health, behavior, feed and water intake, and signs of distress. Environmental conditions, including temperature, ventilation, lighting, and stocking density, were maintained according to Ross 308 management recommendations. Humane endpoints included severe lethargy, inability to access feed or water, persistent recumbency, severe respiratory distress, traumatic injury, or any condition associated with irreversible suffering. Any bird reaching these criteria would have been immediately removed and humanely euthanized. Mortality was not used as an experimental endpoint. All efforts were made to minimize pain and distress. No surgical procedures were performed and no analgesics were required. At the end of the experiment, selected birds were humanely euthanized by carbon dioxide (CO_2_) inhalation (asphyxiation) followed by confirmation of death before carcass evaluation.

### SBM preparation and composition

The SBM used in the present study was prepared according to the method previously reported by Behboodi, Sedaghat [3]. Briefly, SBM consisted of a standardized mixture of betaine, vitamin C, and commercial herbal extracts of *Hypericum perforatum* L. (St John’s wort), lavender, and *Melissa officinalis*, combined to obtain fixed concentrations of each component. The chemical composition of the mixture was characterized using gas chromatography based on the method of Dunford and Vazquez [10]. Amount of the bioactive components including hypericin, linalool, rosmarinic acid, and chlorogenic acid was reported by Behboodi, Sedaghat [3].

### Birds, housing, and experimental design

This experiment used 400 one-day-old mixed-sex Ross 308 broiler chicks randomly assigned to four dietary treatments with five replicate pens of 20 birds each in a completely randomized design. The pen was the experimental unit for all data. Chicks were randomly allocated immediately after arrival, resulting in an approximately equal male:female ratio among treatments. Birds were housed in floor pens bedded with wood shavings and equipped with standard feeders and nipple drinkers, with feed and water supplied ad libitum throughout the 42-day experiment. All birds received a basal corn–soybean meal diet (Table 1) as we used in the series of experiments to assess the drinking additives [11], which was formulated according to Aviagen guidelines [12]. Experimental treatments consisted of supplementation of Stress-Biomax (SBM) in drinking water at four levels: T1 (control, no SBM), T2 (0.25 mL/L), T3 (0.5 mL/L), and T4 (1 mL/L). Supplementation began on day 21 and continued daily until day 42. Supplementation was initiated during the grower phase (day 21), when metabolic demand and oxidative challenge increase rapidly because of accelerated growth. This strategy was chosen to evaluate the physiological effects of SBM during the period of greatest metabolic activity while avoiding unnecessary supplementation during early chick development. The same administration schedule has also been adopted in previous studies evaluating drinking-water phytogenic additives.

**Table 1:** Composition of basal diet (%, as is).

Ingredient	Starter	Grower	Finisher
Corn, Grain	52.50	49.06	52.50
Wheat, Red W.	20.61	15.00	20.61
Soybean Meal	18.98	26.75	18.98
Corn Gluten Meal	2.48	3.73	2.48
Limestone	1.44	1.42	1.44
DCP	1.31	1.47	1.31
Soybean Oil	1.00	1.09	1.00
NaHCO_3_	0.60	0.34	0.60
L-Lysine HCl	0.30	0.29	0.30
Vitamin Premix^1^	0.25	0.25	0.25
Mineral Premix^2^	0.25	0.25	0.25
DL-Methionine	0.15	0.20	0.15
NaCl	0.10	0.10	0.10
L-Thr	0.04	0.04	0.04
Nutrient specifications			
AME (kcal/kg)	2950	2950	2983
CP (%)	23.7	21.8	18.2
SID Lys (%)	1.20	1.10	0.91
SID M + C (%)	0.86	0.79	0.66
SID Thr (%)	0.76	0.69	0.57
SID Met (%)	0.56	0.51	0.41
Ca (%)	1.05	0.95	0.91
Available P (%)	0.50	0.45	0.40
DEB (mEq/kg)^3^	250	250	250

^1^Provided the following per kilogram of diets: vitamin A (trans-retinyl acetate), 3600 IU; vitamin D3 (cholecalciferol), 800 IU; vitamin E (DL-a-tocopheryl acetate), 7.2 mg; vitamin K3, 1.6 mg; thiamine, 0.72 mg; riboflavin, 3.3 mg; niacin, 0.4 mg; pyridoxine, 1.2 mg; cobalamine, 0.6 mg; folic acid, 0.5 mg; choline chloride, 200 mg.

^2^Provided the following per kilogram of diets: Mn (from MnSO4, H 2O), 40 mg; Zn (from ZnO), 40 mg; Fe (from FeSO4.7H2O), 20 mg; Cu (from CuSO4, 5H2O), 4 mg; I [from Ca (IO3)2H2O], 0.64 mg; Se (from sodium selenite), 0.08 mg.

^3^Dieary electrolyte balance (Na+  + K+ - Cl^-^).

### Management program

Birds were reared under standard commercial management practices for Ross 308 broilers, including temperature adjustments in accordance with age-related recommendations, routine vaccination schedules. Lighting was provided using a commercial photoperiod program similar to that described by Behboodi, Sedaghat (3), with gradual changes in light intensity and duration to support normal growth and development.

### Growth performance

Body weight and feed intake were recorded weekly on a replicate basis to calculate average daily gain (G), average daily feed intake (FI), and feed conversion ratio (FCR) for each phase and for the entire 42-day experimental period. Mortalities were monitored daily, and feed intake values were corrected accordingly to ensure accurate performance measurements.

### Internal organ measurements

On day 42, two birds per replicate were randomly selected and individually weighed. Following blood collection, birds were humanely euthanized by carbon dioxide (CO_2_) inhalation and death was confirmed before carcass dissection. Breast and thigh portions were excised and weighed, and their yields (BMY and TMY) were expressed as percentages of live body weight. The abdominal fat pad was removed and weighed using a digital scale with 0.001 g precision.

### Blood sampling and biochemical assays

At 42 days of age, two birds per replicate pen (10 birds per treatment) were randomly selected for blood sampling following a 4-hour fast. Blood samples were collected from the brachial vein and mixed in each replicate pen, centrifuged to obtain serum, and analyzed for lipid profile (TC: total cholesterol, TG: triglycerides, HDL: high-density lipoprotein, LDL: low-density lipoprotein, and VLDL: very low-density lipoprotein), metabolic indices (TP: total protein, ALB: albumin, GLOB: globulin, T-BR: total bilirubin, D-BR: direct bilirubin, I-BR: indirect bilirubin, and GLU: glucose), antioxidant biomarkers (TAC: total antioxidant capacity, MDA: malondialdehyde, SOD: superoxide dismutase, and GPX: glutathione peroxidase), and liver enzymes (ALT: alanine transaminase, AST: aspartate transaminase, and ALP: alkaline phosphatase). Additionally, cortisol and triiodothyronine (T3) concentrations were measured on days 35 and 40 to evaluate stress responses. All biochemical parameters were assessed using commercial diagnostic kits according to manufacturer instructions, consistent with methods reported by Behboodi et al. (2021). Blood collection was performed by trained personnel after a 4-h feed withdrawal. Handling time was minimized to reduce stress and birds were manually restrained for the shortest possible duration. Following blood sampling, birds selected for carcass measurements were euthanized by CO_2_ inhalation according to the approved protocol.

### Statistical analysis

All statistical analyses were performed in R (Version 4.5.1). Data were analyzed under a completely randomized design, and treatment effects across SBM dose levels were first tested using one-way analysis of variance (ANOVA). Statistical significance was declared at *P* < 0.05. To integrate the multivariate biomarker response into a single global indicator, principal component analysis (PCA) was performed on standardized biomarker variables (z-scores) using the *prcomp* function. Variables with an excessive number of missing values or zero variance were excluded prior to analysis. The first principal component (PC_1_) was interpreted as a composite index reflecting the main shared variation among metabolic, oxidative, hepatic, and endocrine variables. A dose–response model was then fitted to PC_1_ using the quadratic and segmented regression models, and the optimal SBM minimizing PC_1_ was identified from model predictions:

(1) Quadratic regression:

$$\begin{array}{c} Y\;=\;\beta_{\text{0}}\;+\;\beta_{\text{1}}\;T\;+\;\beta_{\text{2}}\;T^{\text{2}}\;+\;\varepsilon \end{array}$$

where Y represents the observed response (PCA-derived composite index), T denotes the SBM dose level, *β*_*0*_ is the intercept corresponding to the predicted response at T = 0, *β*_*1*_ is the linear regression coefficient describing the first-order change in Y per unit increase in SBM dose, and *β*_*2*_ represents the quadratic term capturing curvature in the response. The error term ε accounts for unexplained variation, assumed to be independently and normally distributed with mean zero and constant variance. When *β*_*2*_ *≠* 0, the model yields a turning point (optimal dose) at:


$$\begin{array}{c} T_{opt}=\;-\frac{\beta_{\text{1}}}{{\text{2}\beta }_{\text{2}}} \end{array}$$

which corresponds to a minimum response when *β*_*2*_ > 0 and a maximum response when *β*_*2*_ < 0. This approach enables identification of biologically meaningful optimal dose levels where the predicted response is minimized or maximized, depending on the desired optimization direction.

(2) Segmented (piecewise) regression:

$$\begin{array}{c} Y\;=\;L\;+\;U\;\times \;(T_{opt}\;-\;X)\;\times \;(X\;<\;T_{opt})\;+\;V\;\times \;(X\;-\;T_{opt})\;\times \;(X\;>\;T_{opt}) \end{array}$$

where Y is the response, L is the intercept, U and V are slopes of the two segments, and *T*_*opt*_ is the breakpoint. For each response, model adequacy was compared using the root mean squared error (RMSE) and Akaike Information Criterion (AIC), and the model with the lowest RMSE and AIC was selected as the best-supported representation of the dose–response relationship.

## Results

### Performance and carcass characteristics

As shown in Table 2, supplementation with SBM significantly affected growth performance and carcass traits (*P* < 0.001), whereas feed intake was not influenced by treatment (*P* = 0.144, *η²*_p_ = 0.28). Body weight gain increased markedly with supplementation, rising from 2712 g in the control group to a peak of 3082 g at 0.50 mL/L (*η²*_p_ = 0.958), followed by a slight decline at 1.00 mL/L. Feed conversion ratio (FCR) improved significantly, reaching its lowest value (1.62) at 0.50 mL/L (*η²*_p_ = 0.943), with both linear and quadratic effects being significant (*P* < 0.001). Carcass traits were significantly enhanced by Stress-BioMax supplementation. Carcass yield increased to its highest value at 0.50 mL/L (73.9%; *η²*_p_ = 0.985), while thigh meat yield (TMY; *η²*_p_ = 0.985) and breast meat yield (BMY; *η²*_p_ = 1.000) were similarly maximized at this level (22.9% and 27.5%, respectively). In contrast, abdominal fat percentage decreased significantly (*η²*_p_ = 0.999), from 2.48% in the control group to 1.69% at both 0.50 and 1.00 mL/L, indicating a strong reduction in fat deposition with supplementation.

**Table 2:** Analysis of variance of the supplemental Stress-BioMax on performance and carcass attributes of broiler chickens.

Response	Stress-BioMax (mL/L)				SEM	Probablity			*η*²_p_
	0.0	0.25	0.50	1.00		ANOVA	Linear	Quadratic	
Feed intake (g)	5051	5047	4995	5082	25.1	0.144	0.719	0.088	0.28
Gain (g)	2712	2777	3082	2826	14.6	<.001	<.001	<.001	0.958
FCR	1.86	1.82	1.62	1.80	0.01	<.001	<.001	<.001	0.943
Carcass (%)	73.0	72.7	73.9	72.9	0.03	<.001	<.001	<.001	0.985
TMY (%)	22.0	22.1	22.9	22.5	0.02	<.001	<.001	<.001	0. 985
BMY (%)	26.0	26.4	27.5	27.0	0.01	<.001	<.001	<.001	1.000
Fatpad (%)	2.48	2.10	1.69	1.69	0.01	<.001	<.001	<.001	0.999

FCR: feed conversion ratio; TMY: thigh meat yield; BMY: breast meat yield.

### Serum biochemical and physiological variables

Serum biochemical analysis revealed that Stress-BioMax supplementation significantly increased levels of albumin (*η²*_p_ = 0.724), total protein (*η²*_p_ = 0.732), and globulin (*η²*_p_ = 0.516), with the highest values observed at the 0.50 mL/L concentration (*P* < 0.01; Table 3). The lipid profile was markedly changed, as evidenced by significant reductions in TG (*η²*_p_ = 0.970), VLDL (*η²*_p_ = 0.970), LDL (*η²*_p_ = 0.969), and TC (*η²*_p_ = 0.954), alongside a substantial increase in HDL levels (η²_p_ = 0.921, *P* < 0.001). Additionally, supplementation led to significant decreases (*P* < 0.001) in serum glucose (*η²*_p_ = 0.968), bilirubin (*η²*_p_ = 0.984 for total, *η²*_p_ = 0.925 for direct, and *η²*_p_ = 0.920 for indirect), and cortisol levels at days 35 (*η²*_p_ = 0.852) and 40 (*η²*_p_ = 0.936), while triiodothyronine concentrations at days 35 (*η²*_p_ = 0.723) and 40 (*η²*_p_ = 0.948) were significantly elevated.

**Table 3:** Analysis of variance of the supplemental Stress-BioMax on serum biochemical and physiological variables in broiler chickens.

Response	Stress-BioMax (mL/L)				SEM	Probablity			*η*²_p_
	0.0	0.25	0.50	1.00		ANOVA	Linear	Quadratic	
Albumin (g/dL)	2.55	2.74	3.19	2.70	0.07	<.001	0.016	<.001	0.724
TP (g/dL)	5.14	5.68	6.43	5.88	0.14	<.001	<.001	0.001	0.732
Glubolin (g/dL)	2.38	2.94	3.24	3.19	0.17	0.008	0.002	0.088	0.516
TG (mg/dL)	97.7	88.9	64.2	75.1	1.12	<.001	<.001	<.001	0.970
VLDL (mg/dL)	19.5	17.8	12.8	15	0.22	<.001	<.001	<.001	0.970
HDL (mg/dL)	55.3	80.9	93.8	72.9	2.05	<.001	<.001	<.001	0.921
LDL (mg/dL)	131	91.4	40.9	80.3	2.87	<.001	<.001	<.001	0.969
TC (mg/dL)	206	190	148	168	2.41	<.001	<.001	<.001	0.954
Glucose (g/dL)	294	281	225	263	2.34	<.001	<.001	<.001	0.968
T-BR (mg/dL)	1.38	1.04	0.61	0.96	0.02	<.001	<.001	<.001	0.984
D-BR (mg/dL)	0.50	0.39	0.30	0.36	0.01	<.001	<.001	<.001	0.925
I-BR (mg/dL)	0.88	0.66	0.31	0.55	0.03	<.001	<.001	<.001	0.920
CORT_35_ (µg/dL)	6.07	3.94	4.15	5.04	0.18	<.001	0.002	<.001	0.852
CORT_40_ (µg/dL)	10.60	6.50	4.36	8.12	0.30	<.001	<.001	<.001	0.936
T_35_ (ng/mL)	1.48	1.68	1.68	1.65	0.03	<.001	<.001	<.001	0.723
T_40_ (ng/mL)	0.43	0.85	1.18	0.77	0.03	<.001	<.001	<.001	0.948

TP: total protein; TG: triglycerides; VLDL: very low-density lipoprotein; HDL: high-density lipoprotein; LDL: low-density lipoprotein; TC: total cholesterol; T-BR: total bilirubin; D-BR: direct bilirubin; I-BR: indirect bilirubin; CORT_35_: cortisol at d 35; CORT_40_: cortisol at d 40; T_35_: triiodothyronine at d 35; T_40_: triiodothyronine at d 40.

### Hepatic enzyme activity

The activity of hepatic enzymes was significantly modulated by the dietary inclusion of Stress-BioMax (Table 4). There were significant linear and quadratic reductions in the serum activities of AST transaminase (*η²*_p_ = 0.967), ALT (*η²*_p_ = 0.984), and ALP (*η²*_p_ = 0.967) across all supplemented groups (*P* < 0.001). The lowest enzyme activities were consistently recorded in the 0.50 mL/L treatment group.

**Table 4:** Analysis of variance of the supplemental Stress-BioMax on hepatic enzymes in broiler chickens.

Response	Stress-BioMax (mL/L)				SEM	Probablity			*η*²_p_
	0.0	0.25	0.50	1.00		ANOVA	Linear	Quadratic	
AST (U/L)	330	292	226	264	3.52	<.001	<.001	<.001	0.967
ALT (U/L)	40.8	35.9	13.4	23.4	0.676	<.001	<.001	<.001	0.984
ALP (U/L)	2081	1807	779	1573	44.5	<.001	<.001	<.001	0.967

AST: aspartate transaminase; ALT: alanine transaminase; ALP: alkaline phosphatase.

### Antioxidant parameters

Stress-BioMax supplementation significantly bolstered the antioxidant defense system of the broiler chickens (Table 5). The TAC (*η^2^*_p_ = 0.983) and the activities of GPX (*η^2^*_p_ = 0.961) and SOD (*η^2^*_p_ = 0.971) increased significantly in a dose-dependent manner (*P* < 0.001), reaching their maximum values at 0.50 mL/L. Conversely, MDA levels decreased significantly (*η^2^*_p_ = 0.948), with the 0.50 mL/L dose providing the greatest reduction (*P* < 0.001).

**Table 5:** Analysis of variance of the supplemental Stress-BioMax on antioxidant parameters in broiler chickens.

Response	Stress-BioMax (mL/L)				SEM	Probablity			*η*²_p_
	0.0	0.25	0.50	1.00		ANOVA	Linear	Quadratic	
TAC (mmol/L)	2.62	4.81	9.35	6.36	0.16	<.001	<.001	<.001	0.983
GPX (U/L)	245	311	459	346	7.83	<.001	<.001	<.001	0.961
SOD (U/L)	2734	3042	4151	3424	45.4	<.001	<.001	<.001	0.971
MDA (nmol/mL)	5.01	3.75	1.91	3.54	0.13	<.001	<.001	<.001	0.948

TAC: total antioxidant capacity; GPX: glutathione peroxidase; SOD: superoxide dismutase; MDA: malondialdehyde.

### Principal component structure

The first two principal components (PC_1_ and PC_2_) effectively captured the predominant multivariate structure of the dataset. Notably, PC1 explained 80.7% of the variance, whereas PC2 explained 6.5% (Fig 1A). The interpretation of PC_1_ was based on the loading pattern shown in Fig 1A. Variables associated with lipid metabolism (TG, LDL, VLDL, TC), glucose, bilirubin, and liver enzymes exhibited positive loadings, whereas antioxidant biomarkers (TAC, GPX, and SOD) together with albumin and total protein loaded in the opposite direction. Based on these coordinated loading patterns and the established biological roles of these biomarkers, PC_1_ was interpreted as a composite physiological index.

**Fig 1:**
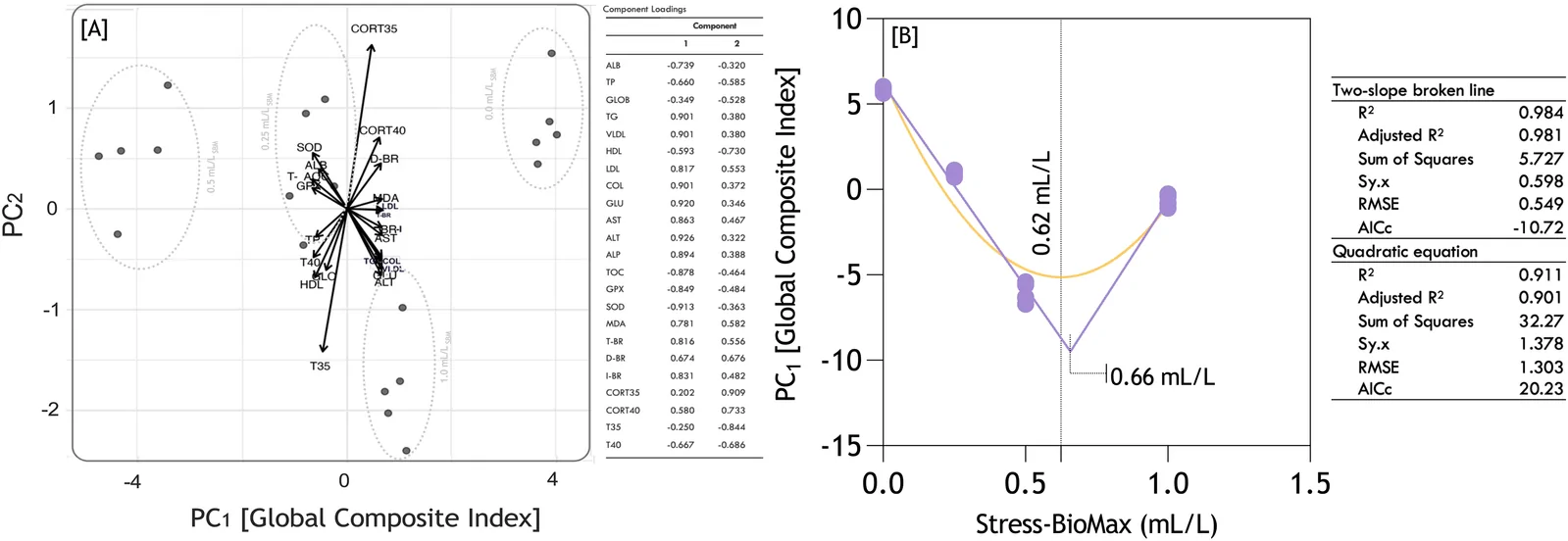
Principal component analysis and dose–response optimization of Stress-BioMax supplementation. (A) Principal component analysis (PCA) biplot showing the loadings of physiological variables and the distribution of experimental samples along PC1 (global composite index) and PC2. Arrows represent variable loadings, and points represent individual experimental units. (B) Dose–response relationship between Stress-BioMax supplementation and the global composite index (PC1). The quadratic regression (solid yellow curve) and two-slope segmented regression (purple broken line) estimated optimal supplementation levels of 0.62 and 0.66 mL/L, respectively. The segmented regression provided the better fit according to model performance statistics (R^2^, RMSE, AICc, and residual error).

#### *PC*_*1*_ *: Global Physiological Index.*

PC1 was driven mainly by higher TG, VLDL, TC, LDL, glucose, hepatic enzymes,bilirubin, and lower TAC, GPX, SOD, albumin, and total protein. This pattern indicates that PC_1_ captures a tightly integrated physiological state defined by dyslipidemia, hyperglycemia, hepatic enzyme leakage, lipid peroxidation, and increased bile pigment turnover. In contrast, TAC and enzymatic antioxidants (GPX and SOD) loaded strongly in the opposite direction, indicating that higher PC_1_ scores correspond to reduced antioxidant defense and greater oxidative strain. ALB and TP also showed moderate negative loadings.

#### *PC*_*2*_ *: Thyroid–Adrenal Stress Axis.*

PC_2_ was dominated by very strong positive loadings of corticosterone (CORT35, CORT40) and strong negative loadings of triiodothyronine (T_3_), indicating a robust inverse relationship between glucocorticoid tone and thyroid activity. Thus, high PC_2_ scores represent a high-corticosterone/low-T_3_ endocrine phenotype, whereas low PC_2_ scores reflect lower corticosterone coupled with higher T_3_ and a more metabolically permissive endocrine state.

### Dose–response modeling of the global physiology index

To quantify the relationship between SBM dose and the global physiological composite response, PC_1_ scores were modeled as a function of dose (Fig 1B). A quadratic regression provided a strong fit to the data (R^2^ = 0.911; adjusted R^2^ = 0.901), indicating a pronounced non-linear dose–response pattern. The quadratic model identified a minimum PC_1_ value at 0.62 mL/L, corresponding to the lowest predicted global physiological composite index.To further evaluate whether the response exhibited a breakpoint structure, a two-slope (segmented) regression model was also fitted. The segmented model showed an even stronger goodness-of-fit (R^2^ = 0.984; adjusted R^2^ = 0.981) and substantially lower residual error (RMSE = 0.549) compared with the quadratic model (RMSE = 1.303). Model comparison using the Akaike Information Criterion (AICc) strongly favored the two-slope model (AICc = −10.72) over the quadratic model (AICc = 20.23), indicating superior explanatory power despite higher model complexity. The segmented regression estimated a breakpoint at 0.66 mL/L, closely aligned with the quadratic minimum, reinforcing the robustness of the identified optimal dose range. Together, these results demonstrate a U-shaped dose–response relationship, with physiological stress being minimized at intermediate Stress-BioMax levels and increasing at both lower and higher doses. The quadratic and segmented regression models produced highly consistent estimates of the optimal SBM supplementation level (0.62 and 0.66 mL/L, respectively), indicating that both modeling approaches converged on the same biologically relevant dose range despite their different mathematical formulations.

## Discussion

### Growth performance

SBM supplementation significantly improved growth performance and carcass traits, as evidenced by increased body weight gain and an improved FCR, while feed intake remained unaffected. The greatest improvement was observed at 0.50 mL/L, suggesting that this intermediate dose supported the best growth efficiency in the present trial. The absence of changes in feed intake confirms that these improvements were driven by enhanced nutrient utilization and metabolic efficiency rather than increased feed consumption. This response pattern is consistent with the significant quadratic effects observed, suggesting a non-linear dose–response relationship in which performance declines slightly at higher inclusion levels.

These findings are in agreement with previous reports demonstrating that phytogenic additives can improve feed efficiency without altering feed intake, primarily through enhancement of digestive function, enzyme activity, and gut health [13,14]. Improved nutrient digestibility and absorption likely contributed to the superior growth performance observed in SBM-supplemented birds. Similar improvements in body weight gain and FCR have been widely reported in broilers receiving phytogenic feed additives, supporting their role as effective growth promoters [15–17].

The enhancement of carcass yield and increases in breast and thigh meat percentages further support the beneficial effects of SBM on productive performance. These responses are consistent with studies showing that phytogenic compounds can promote muscle accretion by modulating protein synthesis pathways and improving nutrient partitioning toward lean tissue deposition [18–20]. Concurrently, the marked reduction in abdominal fat indicates a shift in energy utilization away from lipogenesis and toward muscle growth, likely mediated by improved lipid metabolism and metabolic regulation [21–23]. The slight reduction in performance at the highest SBM dose (1.00 mL/L) suggests diminishing returns at excessive inclusion levels, which is consistent with the hormetic behavior commonly observed for phytogenic compounds. Moderate doses appear to stimulate beneficial metabolic responses, whereas higher doses may impose metabolic constraints or alter nutrient partitioning efficiency. This interpretation is further supported by the dose–response modeling results, which identified an optimal physiological range slightly above the performance peak, emphasizing that maximal growth and minimal composite physiological response do not necessarily coincide.

### Serum biochemistry and metabolic regulation

The observed shifts in serum biochemical variables reflect systemic metabolic improvements associated with SBM supplementation, consistent with reports that blood biochemistry is a sensitive indicator of metabolic status in poultry [24]. Increases in TP, albumin, and globulin indicate enhanced hepatic synthetic capacity and improved protein metabolism, as serum protein fractions are recognized markers of nutritional and immune status in chickens and are often interpreted as signs of improved metabolic and health status in broilers receiving beneficial dietary supplements [24–26].

The lipid profile changes, marked reductions in TG, VLDL, LDL, and TC alongside increases in HDL, highlight a favorable modulation of lipid metabolism, in line with studies where phytogenic additives lowered TC, LDL, and TG while increasing HDL in broilers [27,28]. In poultry, dysregulated lipid profiles can mirror poor metabolic health and predispose birds to excessive fat deposition and oxidative stress [23,27]. Phytogenic bioactives, rich in antioxidants and metabolic modulators, have been shown to influence lipid metabolism, potentially by inhibiting lipogenic enzymes and upregulating pathways involved in lipid oxidation and transport [13,27]. These effects align with reduced abdominal fat deposition seen in SBM groups and suggest that phytogenic compounds in SBM may promote metabolic pathways that favor lean tissue accretion over fat storage [13,23].

SBM supplementation decreased serum glucose, pointing to improvements in carbohydrate metabolism and glycemic control. The reduction in glucose may indicate improved carbohydrate utilization, although insulin sensitivity was not directly measured [27,29]. The reductions in bilirubin (total, direct, and indirect) further suggest improved hepatic function and clearance, which agrees with evidence that phytogenic additives can exert hepatoprotective effects and improve liver enzyme and pigment profiles in birds [27,30]. Bilirubin is often elevated in conditions of hepatic stress or hemolysis; its reduction with SBM supplementation supports the hepatoprotective interpretation further discussed in the hepatic enzyme results [24]. Collectively, these biochemical improvements suggest that SBM positively influences systemic metabolic health beyond growth performance [13,27].

### Hepatic function and enzyme modulation

Assessment of hepatic enzyme activities provides insight into liver health and cellular integrity in poultry [3,31]. The decreases in AST, ALT, and ALP are consistent with improved liver status and reduced hepatocellular leakage. Elevated liver enzyme activities are commonly interpreted as markers of hepatocellular damage or increased membrane permeability; thus, their reduction suggests improved liver integrity and function [3,32]. Modern broiler strains are particularly susceptible to metabolic strain on the liver due to rapid growth, high nutrient throughput, and associated oxidative stress, making hepatic protection especially important [29,33]. Maintenance of hepatic health is critical for optimal performance, as the liver plays central roles in nutrient metabolism, detoxification, and synthesis of key proteins and enzymes [29,34]. The hepatoprotective effects of SBM are likely mediated through antioxidant action (e.g., scavenging of free radicals, reduction of lipid peroxidation) and modulation of inflammatory pathways by phytogenic compounds such as phenolic acids and terpenes, which are known to enhance antioxidant enzyme activity and reduce oxidative damage in poultry liver [34–36]. This protective effect is further supported by biochemical and antioxidant findings discussed below, and aligns with literature documenting the ability of plant-derived additives to reduce hepatic oxidative injury and stabilize hepatocellular membranes [33,37]. Such hepatoprotective effects are crucial for sustaining high growth potential in fast-growing broilers, as even subclinical liver stress can impede nutrient utilization and overall performance [29,34,38].

### Antioxidant defense system strengthening

One of the most robust outcomes of SBM supplementation was the improvement in antioxidant parameters, as reflected by significant increases in TAC and activities of key antioxidant enzymes such as SOD and GPX, accompanied by reductions in MDA, which mirrors reports of phytogenic-supplemented broilers showing higher SOD/GPX and lower MDA [39,40]. Oxidative stress arises when reactive oxygen species (ROS) production exceeds the capacity of endogenous antioxidant defenses, and SOD–GPX systems are central components of this defense network [2,41]. In poultry, high metabolic rates, rapid growth, and intensive production conditions exacerbate ROS generation even in the absence of overt environmental stress, potentially contributing to suboptimal performance and physiological strain [2,40]. By enhancing antioxidant capacity, SBM appears to mitigate chronic oxidative strain, preserving cellular integrity and reducing the biochemical burden associated with ROS accumulation, similar to observations in broilers receiving phytogenic antioxidant blends [40,42]. The phytogenic components of SBM, rich in polyphenolic compounds such as rosmarinic acid and other antioxidant molecules, may contribute to free radical scavenging and support endogenous antioxidant defenses, consistent with evidence that rosmarinic acid improves liver antioxidant activity and decreases MDA in broilers [43,44]. Concurrently, vitamin C provides potent water-soluble antioxidant action, directly neutralizing free radicals and regenerating other antioxidants such as vitamin E, a role widely documented in poultry under various stressors [45,46]. These complementary mechanisms provide both immediate and sustained protection against oxidative damage [40]. The significant reduction in MDA, a marker of lipid peroxidation, indicates that cellular membranes are better preserved in SBM-supplemented birds, which is consistent with improved hepatic integrity and reduced metabolic stress reported when antioxidant or phytogenic supplements lower MDA in poultry tissues [43,47].

### Endocrine responses: Thyroid–adrenal axis

The endocrine results, particularly the reduction in cortisol and the elevation in T_3_, reflect significant modulation of hormonal axes by SBM. Cortisol (corticosterone as the principal glucocorticoid in poultry) is a primary indicator of physiological stress and catabolic activity, and elevated corticosterone levels are associated with increased protein breakdown, impaired immune function, and reduced growth in chickens [48–50]. In the present study, the observed dose-dependent reduction in cortisol suggests that SBM effectively attenuated baseline stress signaling, even in the absence of external stressors, indicating a dampened hypothalamic–pituitary–adrenal (HPA) axis response [51]. By suppressing the HPA axis, the catabolic effects of glucocorticoids will be reduced, preserving muscle protein and fostering muscle growth [48,49]. Conversely, the increase in T_3_, the metabolically active thyroid hormone, indicates enhanced thyroid function and metabolic rate [52]. Thyroid hormones are central regulators of energy metabolism, protein synthesis, and tissue growth in poultry [53,54]. Elevated T_3_ levels in SBM-supplemented groups suggest improved metabolic efficiency and nutrient partitioning toward productive processes [53]. The thyroid–adrenal hormonal balance governs the trade-off between growth and stress adaptation, and SBM appears to shift this balance toward a more growth-permissive phenotype [55]. Mechanistically, glucocorticoid signaling can inhibit thyroid hormone secretion and peripheral conversion of T_4_ to T_3_, leading to suppressed metabolic rates and reduced growth potential [56]. By reducing cortisol levels, SBM may indirectly allow for greater T_3_ production and metabolic activity, supporting enhanced growth performance and carcass traits [53]. These endocrine shifts mirror physiological states of improved growth and metabolic balance and are consistent with studies demonstrating the interplay between stress hormones and growth regulation in poultry [48,57].

### Multivariate integration: Interpretation of PCA results

PCA was applied to the full set of measured physiological variables to derive an integrated index of systemic response to SBM supplementation, providing a multivariate summary of how the additive shapes whole‑body physiology [58]. The PCA suggested that samples diﬀered primarily along PC1 across SBM doses, indicating that treatment effects were expressed primarily as shifts in overall physiological state rather than isolated changes in individual biomarkers, and consistent clustering by dose further supports the suitability of PC_1_ as a synthetic response variable for dose–response modeling [59]. Several stress‑ and metabolism‑related variables exhibited strong loadings on PC_1_, confirming that this axis reflects an integrated physiological response rather than statistical redundancy, in line with prior PCA applications to complex metabolic and hematological profiles in poultry [60,61]. PC_1_ can therefore be interpreted as a composite global physiological index that integrates lipid transport (TG, VLDL, LDL, TC), glycemic control (GLU), antioxidant and redox status (MDA), liver enzyme activity (AST, ALT, ALP), and bilirubin metabolism into a single multivariate score. Animals with high PC_1_ values exhibit a more dysregulated, pro‑oxidative, hepatically challenged phenotype characterized by dyslipidemia, hyperglycemia, elevated oxidative damage, and impaired detoxification, whereas low PC_1_ values reflect a more favorable biochemical and redox profile with lower metabolic burden and stronger antioxidant defense. Albumin and TP showed moderate negative loadings on PC_1_, suggesting that the high‑burden state is accompanied by comparatively lower protein/albumin status, consistent with impaired hepatic synthetic capacity or redistribution of protein resources under physiological stress [25,62].

In contrast, PC_2_ emerged primarily as an endocrine axis rather than a direct liver‑injury component, driven largely by the inverse relationship between corticosterone and T_3_. This negative association between glucocorticoids and thyroid hormone is consistent with stress‑adaptation physiology, where activation of the HPA axis increases glucocorticoid secretion, which can suppress thyroid function via reduced thyroid stimulating hormone (TSH) secretion and inhibition of peripheral T_4_ → T_3_ conversion, ultimately lowering circulating T_3_ [63,64]. Accordingly, PC_2_ may reflect a shift from an anabolic, thyroid‑supported metabolic profile toward a more catabolic, stress‑coping endocrine state under elevated glucocorticoid signaling, reinforcing the interpretation that SBM drives dose‑dependent movement of birds toward a low‑PC_1_/low‑stress, metabolically efficient phenotype.

### Dose–response modeling and optimization

Leveraging the PCA‑derived global physiological index for dose–response modeling revealed a U‑shaped relationship between SBM dose and composite physiological health, illustrating how multivariate indices can capture non‑linear systemic responses to bioactive additives [65,66]. Although univariate performance outcomes pointed to 0.50 mL/L as an effective dose, multivariate modeling identified an optimal range closer to 0.62–0.66 mL/L based on segmentation of PC_1_, underscoring that growth performance alone may not fully describe metabolic burden or resilience [65]. This difference highlights the added value of integrated indices over single parameters, as a multivariate score synthesizes multiple dimensions of physiological function into one interpretable endpoint [8].

The segmented regression model provided a better fit than the simple quadratic model, suggesting that the dose–response relationship may exhibit an approximate biological threshold rather than a simple linear or unimodal pattern. Because only four supplementation levels were evaluated, the estimated breakpoint should be interpreted as an approximate threshold rather than a precise optimum [9]. The slight divergence between growth‑centric and physiology‑centric optimal doses suggests that while 0.50 mL/L maximizes traditional outcomes such as weight gain and FCR, slightly higher doses may further optimize subtle physiological resilience markers without compromising performance [9]. Excessively high doses (e.g., 1.00 mL/L) appeared to plateau or modestly redirect physiological resources, possibly due to the high bioactivity of certain phytochemicals at elevated concentrations, which can differentially modulate metabolic and antioxidant pathways [42,67].

The observed U‑shaped response is consistent with the hormesis framework, in which low to moderate doses of bioactive compounds produce beneficial stimulation, whereas higher doses yield diminishing returns or adverse effects [68,69]. Such hormetic, U‑ or inverted‑U‑shaped dose–response patterns have been repeatedly documented for nutritional and pharmacological agents, including plant‑derived bioactives and antioxidants, with important implications for defining optimal inclusion levels [68].

## Conclusion

SBM supplementation improved growth performance and several physiological biomarkers in broilers, with the most favorable responses generally observed at intermediate doses. These benefits were supported by stronger antioxidant defenses, lower lipid peroxidation, improved lipid and glucose profiles, reduced bilirubin, and decreased liver enzyme activity, alongside a more favorable endocrine state (lower cortisol, higher T_3_). PCA confirmed a dose-dependent shift toward a healthier overall physiological profile, and dose–response modeling revealed a U-shaped pattern, identifying 0.62–0.66 mL/L as the optimal range for minimizing systemic burden. Overall, SBM functions as an effective metabolic stabilizer, and PCA-guided optimization offers a holistic approach for defining the most biologically effective dose in broiler production.

## Supporting information

S1 Data Dataset used for the statistical analyses reported in the manuscript. (XLSX)
