# Supplementary material for: A multivariate PCA-based evaluation of betaine, vitamin C, and phytogenic (STRESS-BIOMax) - supplemented drinking water in broilers?
Source: PLoS One. 2026 Nov 2;21(11):e0357659. doi: 10.1371/journal.pone.0357659 (PMC13727042; doi:10.1371/journal.pone.0357659)
Supplement: Supplementary file 1 [file pone.0357659.s002.docx]

**Response to Editorial Office**

**Comment 1**

We note that your study design may include the death of a regulated animal…

**Response**

We thank the Editorial Office for this comment. The Materials and Methods section has been revised to provide detailed information regarding animal welfare monitoring, humane endpoints, and measures implemented to minimize pain and distress.

**Comment 2**

Please provide additional information regarding methods of sacrifice, anesthesia and/or analgesia, and efforts to alleviate suffering.

**Response**

Additional information has been incorporated into the Ethics Statement, Blood Sampling, and Internal Organ Measurements sections. Birds selected for carcass evaluation were humanely euthanized by carbon dioxide (CO₂) inhalation followed by confirmation of death. Animals were monitored at least twice daily, mortality was not used as an experimental endpoint, and all efforts were made to minimize pain and distress throughout the study.
