# Supplementary material for: A multivariate PCA-based evaluation of betaine, vitamin C, and phytogenic (STRESS-BIOMax) - supplemented drinking water in broilers?
Source: PLoS One. 2026 Nov 2;21(11):e0357659. doi: 10.1371/journal.pone.0357659 (PMC13727042; doi:10.1371/journal.pone.0357659)
Supplement: Supplementary file 2 [file pone.0357659.s003.docx]

**RESPONSE TO REVIEWER**

**Dear Editors,**

We sincerely appreciate the reviewer’s constructive comments, which have substantially improved the clarity, rigor, and interpretation of our study. The revisions include clarification of the study novelty, expansion of the comparison with the previous heat-stress study, improved interpretation and presentation of the PCA results, revision of the dose-response analysis and discussion, clarification of experimental design details, and inclusion of effect size information and study limitations. We believe these changes have substantially strengthened the manuscript. Our detailed responses to each comment are provided below. **All requested changes have been incorporated into the revised manuscript and are highlighted using track changes.**

**Sincerely.**

Mehran Mehri [corresponding author]

Reviewer #1:

This study systematically evaluated the effects of the Stress-BioMax (SBM) supplement in drinking water on broiler growth performance, serum biochemical parameters, antioxidant status, and endocrine responses. It innovatively employed principal component analysis (PCA) and piecewise regression models to determine the optimal supplementation dosage. The experimental design was quite comprehensive, the dataset was abundant, and the application of statistical methods—especially PCA and piecewise regression—demonstrated methodological innovation. However, this manuscript has several issues that require careful attention regarding the novelty claim, the rigor of the results interpretation, and the depth of discussion.

1. Serious Concerns Regarding the Novelty of the Study (Large Overlap with Published Work)

According to the literature, an identical SBM formulation (Stress-Bio-Max, containing St. John's wort, lavender, Melissa officinalis extract, betaine, and vitamin C) was published in the journal *Poultry Science* in 2021 (Poultry Sci. 26 June 2021;100(9):101344. doi:10.1016/j.psj.2021.101344). This published study used the same four treatment groups (0, 0.25, 0.50, 1.00 mL/L), the same Ross 308 strain, the same SBM formulation, and a similar experimental period.

This study reported the beneficial effects of SBM on growth performance, cortisol, AST, ALT, and ALP in broiler chickens under high-temperature stress. Therefore, it is recommended that the authors clearly state the innovative contributions of this study in the introduction. If this experiment was conducted under non-heat stress (normal feeding) conditions, while the 2021 study was conducted under heat stress conditions, this could constitute a meaningful distinction. Even so, this distinction must be clearly stated in the introduction and systematically compared with published results in the discussion.

**Response:** We thank the reviewer for this valuable comment. We have revised both the Introduction and Discussion to clearly distinguish the present study from the previously published work. Specifically, we now emphasize that the previous study evaluated SBM under chronic heat stress, whereas the present study investigated SBM supplementation under normal rearing conditions and focused on the integrated physiological responses of broilers. We also highlight the methodological novelty of combining principal component analysis (PCA) with dose-response optimization to derive a composite physiological index and determine the biologically optimal supplementation level. Furthermore, the Discussion has been expanded to systematically compare our findings with those reported in the previous heat-stress study.

2. The biological interpretation of PC1 is overly simplistic and lacks sufficient support.

The authors interpret PC1 as a "comprehensive indicator of metabolic-oxidative-liver load," believing that a high PC1 value represents a "dysregulation, pro-oxidative, and liver-damaged phenotype." However:

1) The interpretation of PC1 depends on the factor loadings of individual variables, but the manuscript does not provide a complete loading matrix or contribution rate, making it impossible for readers to verify this interpretation.

**Response:** We appreciate the reviewer’s constructive comments. The PCA loading matrix and variable contributions were already presented in the PCA figure.

2) Classifying such a wide range of physiological variables (lipids, glucose, antioxidant enzymes, liver enzymes, bilirubin, etc.) into a single "burden" axis lacks independent biological validation (e.g., histopathological examination, direct measurement of oxidative damage markers).

**Response:** We thank the reviewer for this important observation. We agree that the PCA-derived composite index does not constitute an independently validated biological marker and cannot substitute for direct evidence such as histopathological evaluation or specific oxidative damage biomarkers. The purpose of PCA in this study was to summarize the coordinated variation among multiple physiologically related variables into an integrated multivariate descriptor, thereby facilitating dose-response evaluation. To avoid overinterpretation, the term ‘burden’ has been removed throughout the manuscript and replaced with the more neutral term ‘global physiological index.

3) Does a high PC1 value truly represent an "unfavorable" state, or does it merely reflect differences in metabolic activity? The directionality (good or bad) of the interpretation requires external validation.

Therefore, it is recommended that: a complete PCA loading table (variable name, loading of PC1 and PC2, and contribution rate) be provided in the results; that the "high = poor" directionality be clearly stated as being based on prior knowledge or data-driven inference, supplemented with appropriate references; and that PC1 be more neutrally named "Comprehensive Physiological Response Index" instead of using a term with connotations such as "burden".

**Response:** We thank the reviewer for this thoughtful comment. We agree that the biological interpretation and directionality of PC1 should be presented with appropriate caution. The PCA loading matrix and variable contributions were already included in the PCA figure, allowing readers to evaluate the contribution of each variable to PC1 and PC2. To improve clarity, we have revised the manuscript to explicitly state that the interpretation of higher PC1 values as reflecting a less favorable physiological state is based on the observed loading pattern together with established biological knowledge, as variables with positive loadings (e.g., liver enzymes, glucose, triglycerides, cholesterol, and bilirubin) are widely recognized as indicators of metabolic stress or impaired physiological status, whereas antioxidant biomarkers exhibited opposite loading patterns. We have added appropriate supporting references and now emphasize that this interpretation represents a biologically informed inference rather than direct experimental validation. Furthermore, to avoid implying that PC1 is a validated measure of physiological burden, we have renamed it **global physiological index** throughout the manuscript.

3. Insufficient justification for the choice of piecewise and quadratic regression models. Piecewise regression models have a higher depth of Riesling (4 parameters vs. 3 parameters), and the improvement in R² may simply reflect overfitting rather than a true biological breakpoint structure. Although AICc supports the piecewise model (−10.72 vs. 20.23), the authors have not provided cross-validation or bootstrap test results to verify the predictive stability of the model. Furthermore, the difference between the optimal dose (0.62 vs. 0.66 mL/L) is biologically negligible and insufficient to strongly support the conclusion that the piecewise model is superior. Therefore, it is recommended that the results be supplemented by retaining one excluding cross-validation or bootstrap sampling to verify the robustness of the segmented model; the discussion should explicitly state that both models provide highly consistent optimal dose estimates (0.62–0.66 mL/L), and the actual recommended dose remains 0.50–0.66 mL/L, rather than overemphasizing the superiority of the segmented model.

**Response:** We appreciate the reviewer’s insightful comment. Because the experiment included only four supplementation levels, the breakpoint parameter of the segmented model cannot be estimated with high precision, and resampling-based validation of the breakpoint was not considered sufficiently informative. Instead, we have revised the manuscript to explicitly acknowledge this limitation and to avoid overstating the numerical precision of the segmented model. Importantly, both the quadratic and segmented regressions independently identified a very similar optimal supplementation level (0.62 and 0.66 mL/L, respectively), indicating that the biological conclusion is robust regardless of the modeling approach. Accordingly, we have revised the Discussion to emphasize that the practical supplementation range is approximately **0.50–0.66 mL/L**, rather than a single exact optimum, and that the segmented model should be interpreted as identifying an approximate biological threshold rather than a precise breakpoint.

4. Other Insufficient Experimental Design Details

1) Timing of SBM Treatment Initiation: The method states that "supplementation begins on day 21 and continues daily until day 42." However, why was day 21 chosen as the starting point? Is there any literature or preliminary experimental support for this?

**Response:** We thank the reviewer for this valuable comment. Day 21 was selected as the initiation point for SBM supplementation to evaluate its effects during the grower–finisher phase, when physiological and metabolic demands become more pronounced and responses to nutritional interventions are more readily detected. This supplementation schedule was consistent with our previous study evaluating the same SBM formulation, thereby allowing meaningful comparison between broilers reared under heat-stress and normal environmental conditions. We have clarified this rationale in the revised Materials and Methods.

2) Mixed Sex: The experiment used "mixed sex Ross 308 broiler chicks," but it is not specified whether the sex ratio was balanced across treatment groups. Sex differences may affect growth performance and metabolic parameters and should be considered or addressed in the statistical model.

**Response:** We thank the reviewer for this important comment. Day-old Ross 308 chicks of mixed sex were randomly allocated to the experimental treatments immediately after arrival, which was intended to achieve an approximately equal distribution of males and females among treatment groups. Individual sex was not determined before allocation; therefore, sex was not included as a factor in the statistical analysis. We have clarified this procedure in the Materials and Methods and acknowledged this as a limitation of the experimental design.

3) Sample Size Calculation: No, provide prior sample size calculations (e.g., based on expected effect size, statistical power, and significance level). Authors are advised to carefully address the above issues during major revisions before resubmitting their manuscript.

**Response:** We appreciate the reviewer’s comment. An a priori sample size calculation was not performed because the experiment was designed according to standard replication practices commonly adopted in poultry nutrition research. To address the reviewer’s concern, we conducted a post hoc assessment of treatment effect sizes. The partial eta-squared (η²p) values for the principal treatment effects ranged from **0.948 to 0.983**, indicating exceptionally large treatment effects according to conventional benchmarks. These results demonstrate that the experiment had sufficient statistical sensitivity to detect the observed biological differences. We have acknowledged the absence of an a priori power analysis as a limitation while reporting the observed effect sizes in the revised manuscript.
