# Supplementary material for: A multivariate PCA-based evaluation of betaine, vitamin C, and phytogenic (STRESS-BIOMax) - supplemented drinking water in broilers?
Source: PLoS One. 2026 Nov 2;21(11):e0357659. doi: 10.1371/journal.pone.0357659 (PMC13727042; doi:10.1371/journal.pone.0357659)
Supplement: Peer review history

# Author comment: Author response to Decision Letter 0

**DOI:** 10.1371/journal.pone.0357659.r001

22 Jun 2026

Attachment Submitted filename: Response to Editorial Office.docx

# Peer review: Decision Letter 0

**DOI:** 10.1371/journal.pone.0357659.r002

**Authors:** Ewa Tomaszewska

23 Jul 2026

PONE-D-26-30242

A multivariate PCA-based evaluation of betaine, vitamin C, and phytogenic-supplemented drinking water in broilers

PLOS One

Dear Dr. Mehri,

Thank you for submitting your manuscript to PLOS ONE. After careful consideration, we feel that it has merit but does not fully meet PLOS ONE’s publication criteria as it currently stands. Therefore, we invite you to submit a revised version of the manuscript that addresses the points raised during the review process.

Please submit your revised manuscript by Sep 20 2026 11:59PM. If you will need more time than this to complete your revisions, please reply to this message or contact the journal office at plosone@plos.org. When you're ready to submit your revision, log on to https://www.editorialmanager.com/pone/ and select the 'Submissions Needing Revision' folder to locate your manuscript file.

Please include the following items when submitting your revised manuscript:

- A letter that responds to each point raised by the academic editor and reviewer(s). You should upload this letter as a separate file labeled 'Response to Reviewers'.
- A marked-up copy of your manuscript that highlights changes made to the original version. You should upload this as a separate file labeled 'Revised Manuscript with Track Changes'.
- An unmarked version of your revised paper without tracked changes. You should upload this as a separate file labeled 'Manuscript'.

If you would like to make changes to your financial disclosure, please include your updated statement in your cover letter. Guidelines for resubmitting your figure files are available below the reviewer comments at the end of this letter.

If applicable, we recommend that you deposit your laboratory protocols in protocols.io to enhance the reproducibility of your results. Protocols.io assigns your protocol its own identifier (DOI) so that it can be cited independently in the future. For instructions see: https://journals.plos.org/plosone/s/submission-guidelines#loc-laboratory-protocols. Additionally, PLOS ONE offers an option for publishing peer-reviewed Lab Protocol articles, which describe protocols hosted on protocols.io. Read more information on sharing protocols at https://plos.org/protocols?utm_medium=editorial-email&utm_source=authorletters&utm_campaign=protocols.

As the corresponding author, your ORCID iD is verified in the submission system and will appear in the published article. PLOS supports the use of ORCID, and we encourage all coauthors to register for an ORCID iD and use it as well. Please encourage your coauthors to verify their ORCID iD within the submission system before final acceptance, as unverified ORCID iDs will not appear in the published article. *Only*  the individual author can complete the verification step; PLOS staff *cannot*  verify ORCID iDs on behalf of authors.

We look forward to receiving your revised manuscript.

Kind regards,

Ewa Tomaszewska, DVM Ph.D

Academic Editor

PLOS One

**Journal Requirements:**

When submitting your revision, we need you to address these additional requirements.

1. Please ensure that your manuscript meets PLOS ONE's style requirements, including those for file naming. The PLOS ONE style templates can be found at

https://journals.plos.org/plosone/s/file?id=wjVg/PLOSOne_formatting_sample_main_body.pdf and

https://journals.plos.org/plosone/s/file?id=ba62/PLOSOne_formatting_sample_title_authors_affiliations.pdf

2. Please note that PLOS One has specific guidelines on code sharing for submissions in which author-generated code underpins the findings in the manuscript. In these cases, we expect all author-generated code to be made available without restrictions upon publication of the work. Please review our guidelines at https://journals.plos.org/plosone/s/materials-and-software-sharing#loc-sharing-code and ensure that your code is shared in a way that follows best practice and facilitates reproducibility and reuse.

3. In the online submission form you indicate that your data is not available for proprietary reasons and have provided a contact point for accessing this data. Please note that your current contact point is a co-author on this manuscript. According to our Data Policy, the contact point must not be an author on the manuscript and must be an institutional contact, ideally not an individual. Please revise your data statement to a non-author institutional point of contact, such as a data access or ethics committee, and send this to us via return email. Please also include contact information for the third party organization, and please include the full citation of where the data can be found.

4. If the reviewer comments include a recommendation to cite specific previously published works, please review and evaluate these publications to determine whether they are relevant and should be cited. There is no requirement to cite these works unless the editor has indicated otherwise.

Additional Editor Comments (if provided):

[Note: HTML markup is below. Please do not edit.]

Reviewers' comments:

Reviewer's Responses to Questions

**Comments to the Author**

1. Is the manuscript technically sound, and do the data support the conclusions?

The manuscript must describe a technically sound piece of scientific research with data that supports the conclusions. Experiments must have been conducted rigorously, with appropriate controls, replication, and sample sizes. The conclusions must be drawn appropriately based on the data presented.

Reviewer #1: Partly

**********

2. Has the statistical analysis been performed appropriately and rigorously?

Reviewer #1: Yes

**********

3. Have the authors made all data underlying the findings in their manuscript fully available?

The PLOS Data policy requires authors to make all data underlying the findings described in their manuscript fully available without restriction, with rare exception (please refer to the Data Availability Statement in the manuscript PDF file). The data should be provided as part of the manuscript or its supporting information, or deposited to a public repository. For example, in addition to summary statistics, the data points behind means, medians and variance measures should be available. If there are restrictions on publicly sharing data—e.g. participant privacy or use of data from a third party—those must be specified.

Reviewer #1: Yes

**********

4. Is the manuscript presented in an intelligible fashion and written in standard English?

PLOS ONE does not copyedit accepted manuscripts, so the language in submitted articles must be clear, correct, and unambiguous. Any typographical or grammatical errors should be corrected at revision, so please note any specific errors here.

Reviewer #1: Yes

**********

5. Review Comments to the Author

Please use the space provided to explain your answers to the questions above. You may also include additional comments for the author, including concerns about dual publication, research ethics, or publication ethics. (Please upload your review as an attachment if it exceeds 20,000 characters)

Reviewer #1:

This study systematically evaluated the effects of the Stress-BioMax (SBM) supplement in drinking water on broiler growth performance, serum biochemical parameters, antioxidant status, and endocrine responses. It innovatively employed principal component analysis (PCA) and piecewise regression models to determine the optimal supplementation dosage. The experimental design was quite comprehensive, the dataset was abundant, and the application of statistical methods—especially PCA and piecewise regression—demonstrated methodological innovation. However, this manuscript has several issues that require careful attention regarding the novelty claim, the rigor of the results interpretation, and the depth of discussion.

1. Serious Concerns Regarding the Novelty of the Study (Large Overlap with Published Work)

According to the literature, an identical SBM formulation (Stress-Bio-Max, containing St. John's wort, lavender, Melissa officinalis extract, betaine, and vitamin C) was published in the journal *Poultry Science* in 2021 (Poultry Sci. 26 June 2021;100(9):101344. doi:10.1016/j.psj.2021.101344). This published study used the same four treatment groups (0, 0.25, 0.50, 1.00 mL/L), the same Ross 308 strain, the same SBM formulation, and a similar experimental period.

This study reported the beneficial effects of SBM on growth performance, cortisol, AST, ALT, and ALP in broiler chickens under high-temperature stress. Therefore, it is recommended that the authors clearly state the innovative contributions of this study in the introduction. If this experiment was conducted under non-heat stress (normal feeding) conditions, while the 2021 study was conducted under heat stress conditions, this could constitute a meaningful distinction. Even so, this distinction must be clearly stated in the introduction and systematically compared with published results in the discussion.

2. The biological interpretation of PC1 is overly simplistic and lacks sufficient support.

The authors interpret PC1 as a "comprehensive indicator of metabolic-oxidative-liver load," believing that a high PC1 value represents a "dysregulation, pro-oxidative, and liver-damaged phenotype." However:

1) The interpretation of PC1 depends on the factor loadings of individual variables, but the manuscript does not provide a complete loading matrix or contribution rate, making it impossible for readers to verify this interpretation.

2) Classifying such a wide range of physiological variables (lipids, glucose, antioxidant enzymes, liver enzymes, bilirubin, etc.) into a single "burden" axis lacks independent biological validation (e.g., histopathological examination, direct measurement of oxidative damage markers).

3) Does a high PC1 value truly represent an "unfavorable" state, or does it merely reflect differences in metabolic activity? The directionality (good or bad) of the interpretation requires external validation.

Therefore, it is recommended that: a complete PCA loading table (variable name, loading of PC1 and PC2, and contribution rate) be provided in the results; that the "high = poor" directionality be clearly stated as being based on prior knowledge or data-driven inference, supplemented with appropriate references; and that PC1 be more neutrally named "Comprehensive Physiological Response Index" instead of using a term with connotations such as "burden".

3. Insufficient justification for the choice of piecewise and quadratic regression models. Piecewise regression models have a higher depth of Riesling (4 parameters vs. 3 parameters), and the improvement in R² may simply reflect overfitting rather than a true biological breakpoint structure. Although AICc supports the piecewise model (−10.72 vs. 20.23), the authors have not provided cross-validation or bootstrap test results to verify the predictive stability of the model. Furthermore, the difference between the optimal dose (0.62 vs. 0.66 mL/L) is biologically negligible and insufficient to strongly support the conclusion that the piecewise model is superior. Therefore, it is recommended that the results be supplemented by retaining one excluding cross-validation or bootstrap sampling to verify the robustness of the segmented model; the discussion should explicitly state that both models provide highly consistent optimal dose estimates (0.62–0.66 mL/L), and the actual recommended dose remains 0.50–0.66 mL/L, rather than overemphasizing the superiority of the segmented model.

4. Other Insufficient Experimental Design Details

1) Timing of SBM Treatment Initiation: The method states that "supplementation begins on day 21 and continues daily until day 42." However, why was day 21 chosen as the starting point? Is there any literature or preliminary experimental support for this?

2) Mixed Sex: The experiment used "mixed sex Ross 308 broiler chicks," but it is not specified whether the sex ratio was balanced across treatment groups. Sex differences may affect growth performance and metabolic parameters and should be considered or addressed in the statistical model.

3) Sample Size Calculation: No, provide prior sample size calculations (e.g., based on expected effect size, statistical power, and significance level). Authors are advised to carefully address the above issues during major revisions before resubmitting their manuscript.

**********

6. PLOS authors have the option to publish the peer review history of their article (what does this mean?). If published, this will include your full peer review and any attached files.

If you choose “no”, your identity will remain anonymous but your review may still be made public.

**Do you want your identity to be public for this peer review?**  For information about this choice, including consent withdrawal, please see our Privacy Policy.

Reviewer #1: No

**********

[NOTE: If reviewer comments were submitted as an attachment file, they will be attached to this email and accessible via the submission site. Please log into your account, locate the manuscript record, and check for the action link "View Attachments". If this link does not appear, there are no attachment files.]

To ensure your figures meet our technical requirements, please review our figure guidelines: https://journals.plos.org/plosone/s/figures

You may also use PLOS’s free figure tool, NAAS, to help you prepare publication quality figures: https://journals.plos.org/plosone/s/figures#loc-tools-for-figure-preparation.

NAAS will assess whether your figures meet our technical requirements by comparing each figure against our figure specifications.

# Author comment: Author response to Decision Letter 1

**DOI:** 10.1371/journal.pone.0357659.r003

7 Aug 2026

RESPONSE TO REVIEWER

Dear Editors,

We sincerely appreciate the reviewer’s constructive comments, which have substantially improved the clarity, rigor, and interpretation of our study. The revisions include clarification of the study novelty, expansion of the comparison with the previous heat-stress study, improved interpretation and presentation of the PCA results, revision of the dose-response analysis and discussion, clarification of experimental design details, and inclusion of effect size information and study limitations. We believe these changes have substantially strengthened the manuscript. Our detailed responses to each comment are provided below. All requested changes have been incorporated into the revised manuscript and are highlighted using track changes.

Sincerely.

Mehran Mehri [corresponding author]

Reviewer #1:

This study systematically evaluated the effects of the Stress-BioMax (SBM) supplement in drinking water on broiler growth performance, serum biochemical parameters, antioxidant status, and endocrine responses. It innovatively employed principal component analysis (PCA) and piecewise regression models to determine the optimal supplementation dosage. The experimental design was quite comprehensive, the dataset was abundant, and the application of statistical methods—especially PCA and piecewise regression—demonstrated methodological innovation. However, this manuscript has several issues that require careful attention regarding the novelty claim, the rigor of the results interpretation, and the depth of discussion.

1. Serious Concerns Regarding the Novelty of the Study (Large Overlap with Published Work)

According to the literature, an identical SBM formulation (Stress-Bio-Max, containing St. John's wort, lavender, Melissa officinalis extract, betaine, and vitamin C) was published in the journal *Poultry Science* in 2021 (Poultry Sci. 26 June 2021;100(9):101344. doi:10.1016/j.psj.2021.101344). This published study used the same four treatment groups (0, 0.25, 0.50, 1.00 mL/L), the same Ross 308 strain, the same SBM formulation, and a similar experimental period.

This study reported the beneficial effects of SBM on growth performance, cortisol, AST, ALT, and ALP in broiler chickens under high-temperature stress. Therefore, it is recommended that the authors clearly state the innovative contributions of this study in the introduction. If this experiment was conducted under non-heat stress (normal feeding) conditions, while the 2021 study was conducted under heat stress conditions, this could constitute a meaningful distinction. Even so, this distinction must be clearly stated in the introduction and systematically compared with published results in the discussion.

Response: We thank the reviewer for this valuable comment. We have revised both the Introduction and Discussion to clearly distinguish the present study from the previously published work. Specifically, we now emphasize that the previous study evaluated SBM under chronic heat stress, whereas the present study investigated SBM supplementation under normal rearing conditions and focused on the integrated physiological responses of broilers. We also highlight the methodological novelty of combining principal component analysis (PCA) with dose-response optimization to derive a composite physiological index and determine the biologically optimal supplementation level. Furthermore, the Discussion has been expanded to systematically compare our findings with those reported in the previous heat-stress study.

2. The biological interpretation of PC1 is overly simplistic and lacks sufficient support.

The authors interpret PC1 as a "comprehensive indicator of metabolic-oxidative-liver load," believing that a high PC1 value represents a "dysregulation, pro-oxidative, and liver-damaged phenotype." However:

1) The interpretation of PC1 depends on the factor loadings of individual variables, but the manuscript does not provide a complete loading matrix or contribution rate, making it impossible for readers to verify this interpretation.

Response: We appreciate the reviewer’s constructive comments. The PCA loading matrix and variable contributions were already presented in the PCA figure.

2) Classifying such a wide range of physiological variables (lipids, glucose, antioxidant enzymes, liver enzymes, bilirubin, etc.) into a single "burden" axis lacks independent biological validation (e.g., histopathological examination, direct measurement of oxidative damage markers).

Response: We thank the reviewer for this important observation. We agree that the PCA-derived composite index does not constitute an independently validated biological marker and cannot substitute for direct evidence such as histopathological evaluation or specific oxidative damage biomarkers. The purpose of PCA in this study was to summarize the coordinated variation among multiple physiologically related variables into an integrated multivariate descriptor, thereby facilitating dose-response evaluation. To avoid overinterpretation, the term ‘burden’ has been removed throughout the manuscript and replaced with the more neutral term ‘global physiological index.

3) Does a high PC1 value truly represent an "unfavorable" state, or does it merely reflect differences in metabolic activity? The directionality (good or bad) of the interpretation requires external validation.

Therefore, it is recommended that: a complete PCA loading table (variable name, loading of PC1 and PC2, and contribution rate) be provided in the results; that the "high = poor" directionality be clearly stated as being based on prior knowledge or data-driven inference, supplemented with appropriate references; and that PC1 be more neutrally named "Comprehensive Physiological Response Index" instead of using a term with connotations such as "burden".

Response: We thank the reviewer for this thoughtful comment. We agree that the biological interpretation and directionality of PC1 should be presented with appropriate caution. The PCA loading matrix and variable contributions were already included in the PCA figure, allowing readers to evaluate the contribution of each variable to PC1 and PC2. To improve clarity, we have revised the manuscript to explicitly state that the interpretation of higher PC1 values as reflecting a less favorable physiological state is based on the observed loading pattern together with established biological knowledge, as variables with positive loadings (e.g., liver enzymes, glucose, triglycerides, cholesterol, and bilirubin) are widely recognized as indicators of metabolic stress or impaired physiological status, whereas antioxidant biomarkers exhibited opposite loading patterns. We have added appropriate supporting references and now emphasize that this interpretation represents a biologically informed inference rather than direct experimental validation. Furthermore, to avoid implying that PC1 is a validated measure of physiological burden, we have renamed it global physiological index throughout the manuscript.

3. Insufficient justification for the choice of piecewise and quadratic regression models. Piecewise regression models have a higher depth of Riesling (4 parameters vs. 3 parameters), and the improvement in R² may simply reflect overfitting rather than a true biological breakpoint structure. Although AICc supports the piecewise model (−10.72 vs. 20.23), the authors have not provided cross-validation or bootstrap test results to verify the predictive stability of the model. Furthermore, the difference between the optimal dose (0.62 vs. 0.66 mL/L) is biologically negligible and insufficient to strongly support the conclusion that the piecewise model is superior. Therefore, it is recommended that the results be supplemented by retaining one excluding cross-validation or bootstrap sampling to verify the robustness of the segmented model; the discussion should explicitly state that both models provide highly consistent optimal dose estimates (0.62–0.66 mL/L), and the actual recommended dose remains 0.50–0.66 mL/L, rather than overemphasizing the superiority of the segmented model.

Response: We appreciate the reviewer’s insightful comment. Because the experiment included only four supplementation levels, the breakpoint parameter of the segmented model cannot be estimated with high precision, and resampling-based validation of the breakpoint was not considered sufficiently informative. Instead, we have revised the manuscript to explicitly acknowledge this limitation and to avoid overstating the numerical precision of the segmented model. Importantly, both the quadratic and segmented regressions independently identified a very similar optimal supplementation level (0.62 and 0.66 mL/L, respectively), indicating that the biological conclusion is robust regardless of the modeling approach. Accordingly, we have revised the Discussion to emphasize that the practical supplementation range is approximately 0.50–0.66 mL/L, rather than a single exact optimum, and that the segmented model should be interpreted as identifying an approximate biological threshold rather than a precise breakpoint.

4. Other Insufficient Experimental Design Details

1) Timing of SBM Treatment Initiation: The method states that "supplementation begins on day 21 and continues daily until day 42." However, why was day 21 chosen as the starting point? Is there any literature or preliminary experimental support for this?

Response: We thank the reviewer for this valuable comment. Day 21 was selected as the initiation point for SBM supplementation to evaluate its effects during the grower–finisher phase, when physiological and metabolic demands become more pronounced and responses to nutritional interventions are more readily detected. This supplementation schedule was consistent with our previous study evaluating the same SBM formulation, thereby allowing meaningful comparison between broilers reared under heat-stress and normal environmental conditions. We have clarified this rationale in the revised Materials and Methods.

2) Mixed Sex: The experiment used "mixed sex Ross 308 broiler chicks," but it is not specified whether the sex ratio was balanced across treatment groups. Sex differences may affect growth performance and metabolic parameters and should be considered or addressed in the statistical model.

Response: We thank the reviewer for this important comment. Day-old Ross 308 chicks of mixed sex were randomly allocated to the experimental treatments immediately after arrival, which was intended to achieve an approximately equal distribution of males and females among treatment groups. Individual sex was not determined before allocation; therefore, sex was not included as a factor in the statistical analysis. We have clarified this procedure in the Materials and Methods and acknowledged this as a limitation of the experimental design.

3) Sample Size Calculation: No, provide prior sample size calculations (e.g., based on expected effect size, statistical power, and significance level). Authors are advised to carefully address the above issues during major revisions before resubmitting their manuscript.

Response: We appreciate the reviewer’s comment. An a priori sample size calculation was not performed because the experiment was designed according to standard replication practices commonly adopted in poultry nutrition research. To address the reviewer’s concern, we conducted a post hoc assessment of treatment effect sizes. The partial eta-squared (η²p) values for the principal treatment effects ranged from 0.948 to 0.983, indicating exceptionally large treatment effects according to conventional benchmarks. These results demonstrate that the experiment had sufficient statistical sensitivity to detect the observed biological differences. We have acknowledged the absence of an a priori power analysis as a limitation while reporting the observed effect sizes in the revised manuscript.

Attachment Submitted filename: response to REV.docx

# Peer review: Decision Letter 1

**DOI:** 10.1371/journal.pone.0357659.r004

**Authors:** Ewa Tomaszewska

20 Aug 2026

A multivariate PCA-based evaluation of betaine, vitamin C, and phytogenic-supplemented drinking water in broilers

PONE-D-26-30242R1

Dear Dr. Mehran Mehri,

We’re pleased to inform you that your manuscript has been judged scientifically suitable for publication and will be formally accepted for publication once it meets all outstanding technical requirements.

Within one week, you’ll receive an e-mail detailing the required amendments. When these have been addressed, you’ll receive a formal acceptance letter and your manuscript will be scheduled for publication.

An invoice will be generated when your article is formally accepted. Please note, if your institution has a publishing partnership with PLOS and your article meets the relevant criteria, all or part of your publication costs will be covered. Please make sure your user information is up-to-date by logging into Editorial Manager at Editorial Manager® and clicking the ‘Update My Information' link at the top of the page. For questions related to billing, please contact billing support.

If your institution or institutions have a press office, please notify them about your upcoming paper to help maximize its impact. If they’ll be preparing press materials, please inform our press team as soon as possible -- no later than 48 hours after receiving the formal acceptance. Your manuscript will remain under strict press embargo until 2 pm Eastern Time on the date of publication. For more information, please contact onepress@plos.org.

Kind regards,

Ewa Tomaszewska, DVM Ph.D

Academic Editor

PLOS One

Additional Editor Comments (optional):

Reviewers' comments:

Reviewer's Responses to Questions

**Comments to the Author**

Reviewer #1: All comments have been addressed

**********

2. Is the manuscript technically sound, and do the data support the conclusions?

Reviewer #1: Yes

**********

3. Has the statistical analysis been performed appropriately and rigorously?

Reviewer #1: Yes

**********

4. Have the authors made all data underlying the findings in their manuscript fully available?

The PLOS Data policy

Reviewer #1: Yes

**********

5. Is the manuscript presented in an intelligible fashion and written in standard English?

Reviewer #1: Yes

**********

Reviewer #1: (No Response)

**********

what does this mean?). If published, this will include your full peer review and any attached files.

If you choose “no”, your identity will remain anonymous but your review may still be made public.

**Do you want your identity to be public for this peer review?**  For information about this choice, including consent withdrawal, please see our Privacy Policy

Reviewer #1: No

**********

# Editor report: Acceptance letter

**DOI:** 10.1371/journal.pone.0357659.r005

**Authors:** Ewa Tomaszewska

PONE-D-26-30242R1

PLOS One

Dear Dr. Mehri,

I'm pleased to inform you that your manuscript has been deemed suitable for publication in PLOS One. Congratulations! Your manuscript is now being handed over to our production team.

At this stage, our production department will prepare your paper for publication. This includes ensuring the following:

* All references, tables, and figures are properly cited

* All relevant supporting information is included in the manuscript submission,

* There are no issues that prevent the paper from being properly typeset

You will receive further instructions from the production team, including instructions on how to review your proof when it is ready. Please keep in mind that we are working through a large volume of accepted articles, so please give us a few days to review your paper and let you know the next and final steps.

Lastly, if your institution or institutions have a press office, please let them know about your upcoming paper now to help maximize its impact. If they'll be preparing press materials, please inform our press team within the next 48 hours. Your manuscript will remain under strict press embargo until 2 pm Eastern Time on the date of publication. For more information, please contact onepress@plos.org.

You will receive an invoice from PLOS for your publication fee after your manuscript has reached the completed accept phase. If you receive an email requesting payment before acceptance or for any other service, this may be a phishing scheme. Learn how to identify phishing emails and protect your accounts at https://explore.plos.org/phishing.

If we can help with anything else, please email us at customercare@plos.org.

Thank you for submitting your work to PLOS One and supporting open access.

Kind regards,

PLOS One Editorial Office Staff

on behalf of

Professor Ewa Tomaszewska

Academic Editor

PLOS One
